# Multi-label remote sensing classification with self-supervised gated multi-modal transformers

**DOI:** 10.3389/fncom.2024.1404623

**Published:** 2024-09-24

**Authors:** Na Liu, Ye Yuan, Guodong Wu, Sai Zhang, Jie Leng, Lihong Wan

**Affiliations:** ^1^University of Shanghai for Science and Technology, Institute of Machine Intelligence, Shanghai, China; ^2^Origin Dynamics Intelligent Robot Co., Ltd., Zhengzhou, China

**Keywords:** self-supervised learning, pre-training, vision transformer, multi-modal, gated units

## Abstract

**Introduction:**

With the great success of Transformers in the field of machine learning, it is also gradually attracting widespread interest in the field of remote sensing (RS). However, the research in the field of remote sensing has been hampered by the lack of large labeled data sets and the inconsistency of data modes caused by the diversity of RS platforms. With the rise of self-supervised learning (SSL) algorithms in recent years, RS researchers began to pay attention to the application of “pre-training and fine-tuning” paradigm in RS. However, there are few researches on multi-modal data fusion in remote sensing field. Most of them choose to use only one of the modal data or simply splice multiple modal data roughly.

**Method:**

In order to study a more efficient multi-modal data fusion scheme, we propose a multi-modal fusion mechanism based on gated unit control (MGSViT). In this paper, we pretrain the ViT model based on BigEarthNet dataset by combining two commonly used SSL algorithms, and propose an intra-modal and inter-modal gated fusion unit for feature learning by combining multispectral (MS) and synthetic aperture radar (SAR). Our method can effectively combine different modal data to extract key feature information.

**Results and discussion:**

After fine-tuning and comparison experiments, we outperform the most advanced algorithms in all downstream classification tasks. The validity of our proposed method is verified.

## 1 Introduction

In recent years, ViT (Dosovitskiy et al., 2020) architecture has been widely studied. It is an attention-based encoder composed of multiple transformers layers stacked together, acting like a backbone network in a convolutional neural network, and is trained to extract the feature representation of input data. Usually, there is a decoder behind the encoder to output of the feature task. ViT is increasingly used in computer vision due to its accuracy, efficiency, and scalability, and delivers state-of-the-art (SOTA) performance for most computer vision (CV) tasks (He et al., 2022; Carion et al., 2020; Peng et al., 2022; Xu et al., 2022; Liu et al., 2022).

**Figure 1:**
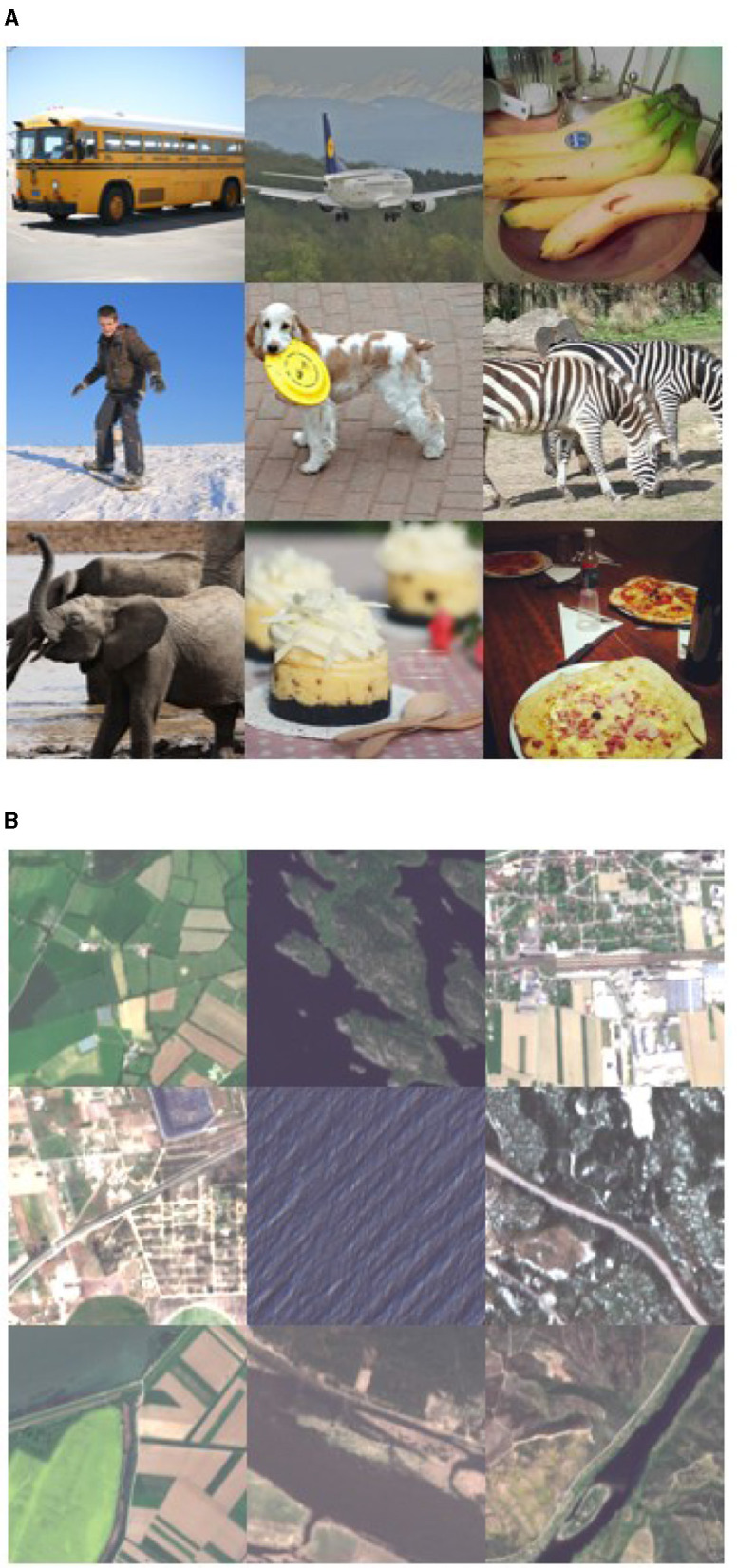
Illustration of the difference between object-centric natural images [from the COCO (Lin et al., 2014) dataset] and remote sensing scene images [from the BigEarthNet (Sumbul et al., 2019) dataset]. **(A)** Object-centric image samples. **(B)** Remote sensing image samples.

The recent success of ViT in CV has made it widely studied in RS as well. As we all know, it is very difficult to obtain labeled data sets in the field of RS, and there are very large data without labels. Therefore, it is suitable to utilize transfer learning in RS (Liang et al., 2019). Transfer learning accelerates and improves learning and problem-solving in a new field by transferring knowledge and experience from one field to another related field, so transfer learning has shown excellent generalization ability in several fields (Pires de Lima and Marfurt, 2019; Shin et al., 2016). However, there are very few pre-trained models dedicated to RS migration, and transfer learning from models trained in other fields [e.g. ImageNet (Russakovsky et al., 2015)] has significant drawbacks. For scene classification, if the pre-training stage is performed on a large remote sensing dataset instead of traditional ImageNet, transfer learning will be more meaningful because the pre-training stage on a large remote sensing dataset helps to provide more meaningful features (Neumann et al., 2020). In fact, the samples from the ImageNet dataset have completely different features from remote sensing images. The former is usually centered around the image, while the latter's specific category features typically exist throughout the entire image (see Figure 1). And we know there are various types of sensors in RS, in addition to containing RGB three optical band data, there are additional bands in the invisible part of the electromagnetic spectrum. In order to better evaluate and compare the performance of remote sensing scenario classification methods, the remote sensing community is striving to create and collect a variety of data sets for benchmarking. These involve different datasets, from simple three channel RGB images to multispectral, hyperspectral, or time series datasets. On the other hand, efforts are also being made to train pre-trained models specifically for RS on RS datasets (Jain et al., 2021; Yuan and Lin, 2021; Vincenzi et al., 2021; Kang et al., 2021).

**Figure 2:**
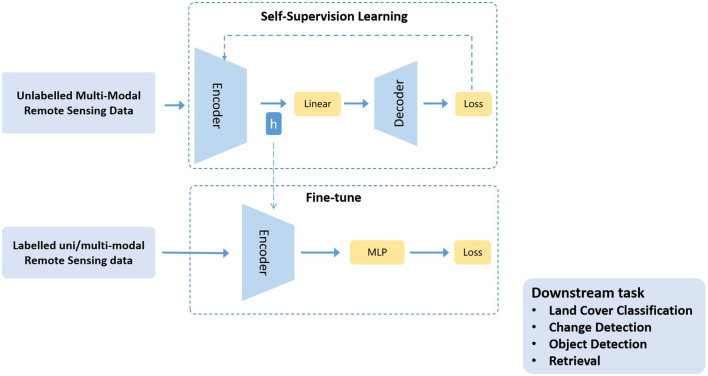
General SSL representation learning approach utilizes unlabeled multi-modal data in RS domain. After pre-training, projection layers are removed and encoder weights are transferred for task specific supervised learning. *h* means initialize encoder with learned representations.

SSL has the ability to learn common representations from large-scale, unlabeled data, which is generally broken down into two main steps: (1) Training a model with unlabeled data to learn common features in the data based on self-supervised goals; (2) Transferring a pre-trained model to a supervised downstream task to leverage its ability to capture good representations. In most common SSL architectures, encoders are trained along with the projection layer, and after training, encoder weights are used with a task-specific classifier or decoder, as shown in Figure 2. Research (Abnar et al., 2021; Zhai et al., 2022; Alabdulmohsin et al., 2022; Tay et al., 2022; Kaplan et al., 2020; Tao et al., 2020; Stojnic and Risojevic, 2021) has shown that the more datasets used for pre-training, the more accurate the pre-trained model is, and the greater its contribution to downstream tasks, which can to some extent solve the problem of RS annotation data scarcity. So in recent years, pre-trained ViT models based on RS data have achieved SOTA performance on some RS tasks (Wang et al., 2022a; Scheibenreif et al., 2022; Wang D. et al., 2022; Cong et al., 2022).

## 2 Related work

In RS, images are acquired through various sensors, the pre-trained datasets for SSL usually contain data from multiple modalities, the two most popular ones currently being MS and SAR. MS remote sensing system records the weak difference of spectral reflection and radiation characteristics of ground objects in different bands, has high spectral resolution, can provide a lot of information such as surface material composition, help to identify different types of ground objects, and is the most important information source for remote sensing application research at present. However, the multi-spectral remote sensing system is vulnerable to the negative impact of weather conditions (such as cloud cover) and has high requirements on weather. Due to the influence of cloud cover and cloud shadow, its data can only be effectively used in sunny days without clouds, which greatly limits the application of its data.

Radar data is an active remote sensing system independent of sunlight. Radar beams can penetrate clouds, not affected by day, night and cloud factors, and obtain data all day and all weather, which just makes up for the lack of MS remote sensing. More importantly, radar information provides physical characteristics of ground objects by reflecting their surface roughness, which mainly depends on the geometric shape of the target, the surface structure trace, such as plant cover, loose sediment (sand, gravel, pebble), etc. Due to these characteristics of radar, it has great application potential in geological structure, topography, surface moisture, soil moisture, vegetation cover, settlements, linear features, sea state recognition and other aspects, but SAR cannot provide easy to interpret images, each modal data has its own advantages and disadvantages. In order to make full use of data information, researchers usually need to use multiple modal data in combination. Moreover, recent studies have shown that simply learning the joint features of the two modes also shows significant advantages (Tao et al., 2020; Vincenzi et al., 2021).

In multi-modal data fusion training, Wang et al. (2022a) proposed a joint SAR-optical SSL algorithm. The concatenated SAR-optical image is taken as raw input. It is randomly transformed into two augmented views and fed into a DINO-based (Caron et al., 2021) teacher-student network. This way, the model contrasts views of either a single modality or both, learning both inner-modality and inter-modality representations. Jain et al. (2022) applied the distillation network concept to build and analyze single channel and three channel features learning for MS and SAR data, utilized MS and SAR data as an implicit augmentation to learn invariant feature embeddings and verified the usefulness of multiple modes for feature learning based on self-supervised distillation network BYOL (Grill et al., 2020). Fuller et al. (2022) pre-trained the ViT model on 1.3 million Sentinel-1 and Sentinel-2 images using a SOTA self-supervised learning algorithm called Mask Automatic coding (He et al., 2022) (MAE). They then loaded the pre-trained model (Fuller et al., 2022) and reduced the patch size to retain finer grained information, achieving better results (Green et al., 2022). Wang et al. (2022b) expose a multi-modal, multitime unlabeled dataset SSL4EO-S12 for Earth observation-like self-supervised learning and validate the advantages of multimodality in multiple downstream tasks using a series of SSL algorithms. These studies show that the multi-modal fusion of MS and SAR images has significant advantages over the use of a single mode. However, in the current research on multi-modal fusion, most of the processed data or extracted features are simply spliced together without considering the proportion of importance of different modes in different tasks. For example, the red-edge band is good at detecting vegetation and soil, and the SWIR and NIR bands are good at detecting water and resolving thin clouds. SAR, on the other hand, is not affected by weather and can help with terrain or disaster-related tasks. Therefore, in the multi-mode fusion, the importance of different modes in different tasks should be fully considered.

In order to solve the problem of multi-modal fusion, we propose a multi-modal gated fusion training method based on self-supervision. In detail, on the basis of the traditional ViT structure, we added the shortcut layer gated fusion mechanism, and the feature vectors extracted from different modes were gated fusion. In the self-supervised training method, we integrated contrast learning based on the MAE method to further improve the performance of the model.

## 3 Proposed method

We propose a self-supervised learning method for multi-modal gated fusion in the field of remote sensing. The traditional method simply splines two data features when SAR and MS multi-modal data fusion. Compared with the traditional method, we use a gated unit to achieve feature fusion of multi-modal data. The intra-modal gating unit is used to control the fusion of different transformers layers. After extracting the feature representation, the inter-modal gating unit fuses the features of different modes to obtain the effective feature representation based on different modes. At the same time, we redesigned a new self-supervised training model based on the mask reconstruction self-supervised method and momentum contrast self-supervised method. Firstly, the self-supervised pre-training was carried out on different modal data, and then in the downstream task, the pre-training models of different modes were loaded, and the model was fine-tune with the inter-modal gated fusion unit.

In detail, suppose we have a multi-modal data set **S** that consists of *N* number of SAR and MS pairs, where $S={\left\{S_{1},S_{2}\right\}}^{N}$ where *S*_1_ represents SAR data that contains 2 channels and *S*_2_ represents MS data that contains 12 channels. Firstly, two pre-trained models *S*_1_ encoder and *S*_2_ encoder are self-supervised trained based on *S*_1_ and *S*_2_ respectively. In the downstream task, the trained pre-trained model is loaded respectively for data feature extraction, and the two groups of extracted feature vectors are gated and fused, and then sent to the classification head for output results.

### 3.1 Vision transformers

We selected the ViT model as the primary architecture for our model. ViT processes feature vectors comprised of a sequence of patches, which are then passed through multiple transformer layers for feature extraction. Each Transformer layer consists of a multi-headed self-attention (MHSA) sublayer and a feed-forward network (FFN) sublayer. MHSA focuses on extracting significant features between patches, while FFN emphasizes significant features within patches. The number of stacked transformer layers in ViT is known as the model depth, and the length of each hidden features is termed the model width. Generally, the model depth and width determine the model's performance. Recognizing that features extracted from shallow transformers may be somewhat forgotten as the model depth increases, we introduced a Transformer-shortcut sublayer (TS) after stacking N transformer layers. The entire ViT model structure comprises multiple TS structures, and introduced a gating unit to control the weight coefficient of the feature vector. This gating unit integrates ideas from feature fusion and decision fusion.

**Figure 3:**
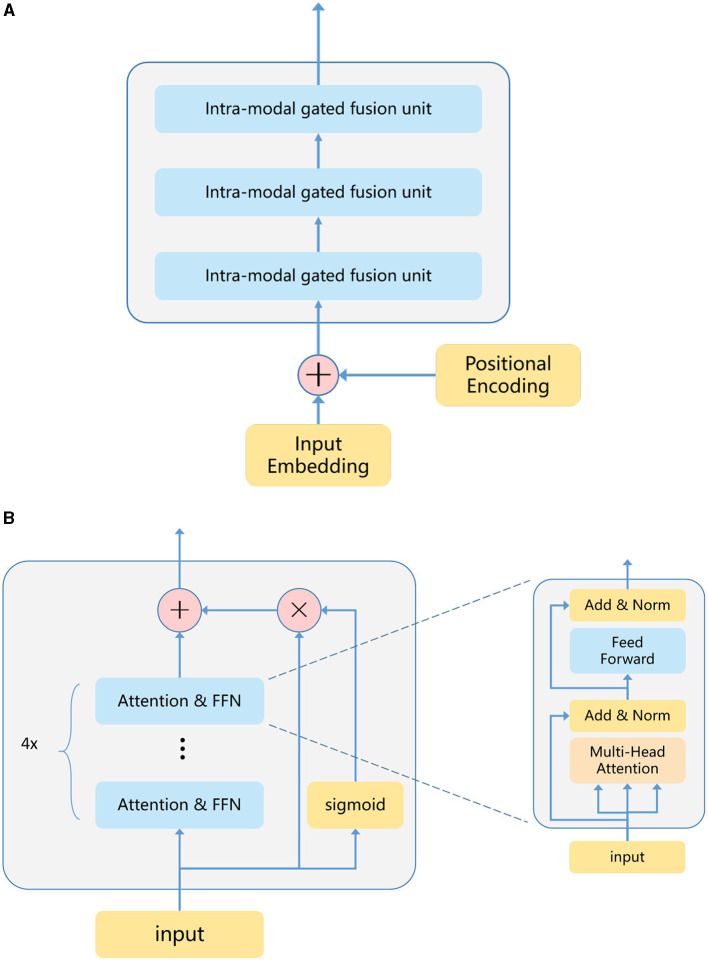
In the encoder diagram proposed by us, the encoder has 12 layers of transformers, which is divided into three inter-modal gated fusion units, and each fusion of the modal internal gating unit is carried out every four transformers layers. **(A)** Encoder. **(B)** Intra-modal gated fusion unit.

In detail, suppose a transformer layer named *H*_*n*_, on the one hand, it will output *H*_*n*+4_ after passing through four layers of transformer, on the other hand, it will output weight coefficient through a linear layer *F* and a *sigmoid* function. The weight multiplied by the *H*_*n*_ layer and directly added to the *H*_*n*+4_ layer as the final output result of the gated unit, Figure 3 shows the gated unit transformer architecture our proposed, and the calculation process is as follows:


(1) $$\begin{array}{l} H_{out}=H_{n+i}+\sigma \left(F\left(H_{n}\right)\right)\times H_{n} \end{array}$$

Here *i* is shortcut number, σ is *sigmoid* function.

We designed the model depth of the ViT encoder to be 12 and the model width to be 768. Before the input data is sent to encoder for encoding, it will be divided into small patches, and then rearranged into a series of patches in sequence. After linear projection of the original pixel value to the 768 underlying features of each patch, positional coding information will be added, which is unique to ViT. They mainly record the position information of each patch in the encoder. The coded patches are then processed by a series of transformer layers. In detail, take *S*1 data as an example, the dimensions of the original *S*1 data are {224, 244, 2}. Firstly, we will divide the input data *S*1 into patches, the dimensions of which are designed to be 16 × 16 pixels, and the dimensions of each patch are {16, 16, 2}. The number of patches after segmentation is 14 × 14, and the dimensions of patches after rearrangement with location coding are {196, 16 × 16 × 2}, and the dimensions of the same *S*2 data after encoding are {196, 16 × 16 × 12}. In transformer coding, feature vector gating fusion will be carried out every four layers, and the model depth is 12, so a total of three times gating fusion will be carried out. The number of transformer layers through which gating fusion is carried out can be changed. After testing, we find that every four layers has the best effect.

### 3.2 Multi-modal fusion

**Figure 4:**
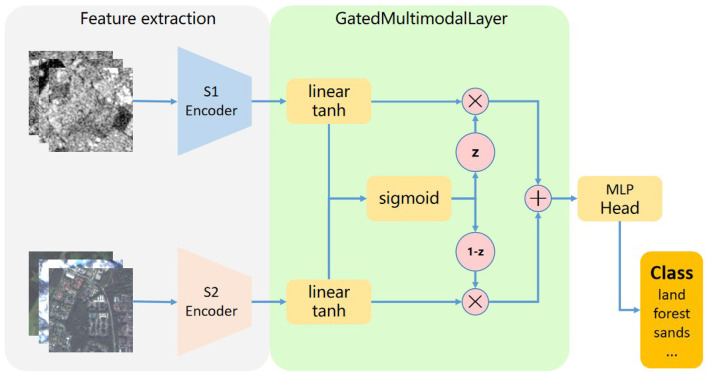
Illustration of gated units. The features of different modal data are extracted by different encoders, fused by gating units, and fed into the classification head network for classification tasks.

In this paper, we introduce a gated multi-modal unit (Arevalo et al., 2017) (GMU) that is similar to the way a cyclic model controls the flow of information, learning how to use multiplicative gates to determine how the modes affect the activation of the unit, the GMU receives two or more input sources, and learns to determine how much each input mode influences the activation of the unit. It combines ideas from feature fusion and decision fusion, and its aim is to find an intermediate representation based on combinations of features of different patterns. The Figure 4 describes the structure of GatedMultimodalLayer. After the two modal data are encoded by encoder respectively, the feature vector of the modal association is obtained. Each mode associated feature vector connects a linear layer with a *tanh* activation function designed to encode an internal representation feature based on a particular mode. For each modal associated feature vector, there will also be a gate neuron (represented by the *sigmoid* node in the figure), which controls the contribution of the features of the different modes to the overall output of the unit. In detail, we assume that *S*1 data is encoded by *S*1 encoder to obtain the feature vector *x*_*s*1_, it passes through the linear layer *W*_*s*1_ and *tanh* activation functions to get the feature representation *h*_*s*1_, *S*2 data is encoded by *S*2 encoder to obtain the feature vector *x*_*s*2_, it passes through the linear layer *W*_*s*2_ and *tanh* activation functions to get the feature representation *h*_*s*2_, the *h*_*s*1_ and *h*_*s*2_ feature vectors then pass through the linear layer *W*_*z*_ and the gating unit *sigmoid* to obtain the gating vector *z*, which then coordinates with *h*_*s*1_ and *h*_*s*2_ to obtain the final feature vector *h*. the entire GMU calculation process is as follows:


(2) $$\begin{array}{l} h_{s1}=\tanh\left(W_{s1}\times x_{s1}\right) \end{array}$$

(3) $$\begin{array}{l} h_{s2}=\tanh\left(W_{s2}\times x_{s2}\right) \end{array}$$

(4) $$\begin{array}{l} z=\sigma \left(W_{z}\times \left[x_{s1},x_{s2}\right]\right) \end{array}$$

(5) $$\begin{array}{l} h=z\times h_{s1}+\left(1-z\right)\times h_{s2} \end{array}$$

After the GMU unit outputs the final feature vector *h*, it maximizes the useful features according to the contribution of different modal features to the overall output, and then carries out the classification task with the classification head. Since all operations of the control unit are differentiable operations, the model can be easily coupled with other neural network architectures and trained by stochastic gradient descent.

### 3.3 Self-supervised model pre-training

In this paper, we pre-trained SAR and MS ViT models respectively. In order to train the encoder of the ViT model, we refer to MAE algorithm, which is currently in the leading position in the self-supervised learning algorithm fetching. The main idea of MAE is to hide most of the position information in the original data, the proportion is as high as 75, and then an asymmetric coding-decoding structure is proposed. encoder only operates on a subset of the unmasked patch part. The image is then reconstructed from hidden space and mask token via a lightweight decoder. In this paper, we first pre-process (e.g. the size is unified to 128 × 128) all the original data. After loading the data, different data enhancement methods are randomly selected to generate other two sets of data, and MAE training will be conducted on these two sets of data respectively. Before the training MAE is sent to the model encoder, the input data will be sorted by patches and 75 patches will be randomly shielded. For patches that are not hidden, the patches will be sent to the encoder for feature extraction processing, and then the patches encoding will be sent to the decoder to reconstruct the corresponding input data information. We set the decoder depth to 2 and width to 384 (length of feature vector per patch).

In detail, we assume that we first pre-process the original data as *x*, then we use different data enhancement methods to generate other two sets of data *x*_1_ and *x*_2_, we randomly shield 75 patches on these two sets of data respectively, we called *m*_1_ and *m*_2_, then they will be sent to the encoder and decoder to reconstruct the corresponding feature respectively, we called *r*_1_ and *r*_2_, when we training encoder, we need to calculate the reconstruction loss of input features and corresponding reconstruction features. The reconstruction loss we used was the mean squared error between predicted and target pixels in the masked patches, we called *MSE*, the reconstruction loss *Loss*_*rec*_ is as follows:


(6) $$\begin{array}{l} loss_{r1}=MSE\left(x_{1},r_{1}\right)\times m_{1} \end{array}$$

(7) $$\begin{array}{l} loss_{r2}=MSE\left(x_{2},r_{2}\right)\times m_{2} \end{array}$$

(8) $$\begin{array}{l} Loss_{rec}=loss_{r1}+loss_{r2} \end{array}$$

In order to further improve the feature extraction capability of the encoder, we refer to the contrast learning idea in the self-supervised learning algorithm and introduce the Momentum contrast (Chen et al., 2021) (MoCoV3) method. Contrast SSL trains the model by encouraging the representation of enhanced versions of the same image to be similar (positive) while contrasting with other images (negative), which aims to align the different image enhanced representations between the model and the momentum encoder, which is a copy of the model updated by the exponential moving average (EMA). Since MAE has been randomly enhanced from the original data to generate two sets of new data *x*_1_ and *x*_2_ during training, during MoCoV3 training, *x*_1_ is sent to Encoder for encoding, *x*_*p*1_ is output through prediction layer, and *x*_*m*1_ is output through momentum encoder layer, and the same process for *x*_2_ to generate *x*_*p*2_ and *x*_*m*2_. The dimension of the output hidden features of the prediction layer is conssistent with that of the momentum encoder, and the contrast loss of the two is calculated. The contrast loss we use is InfoNCE (Oord et al., 2018), the contrast loss *Loss*_*con*_ is as follows:


(9) $$\begin{array}{l} Loss_{con}=InfoNCE\left(x_{p1},x_{m2}\right)+InfoNCE\left(x_{p2},x_{m1}\right) \end{array}$$

After calculating the reconstruction loss and the contrast loss, we can get the final loss, the final loss *Loss* is as follows:


(10) $$\begin{array}{l} Loss=Loss_{rec}+Loss_{con} \end{array}$$

**Figure 5:**
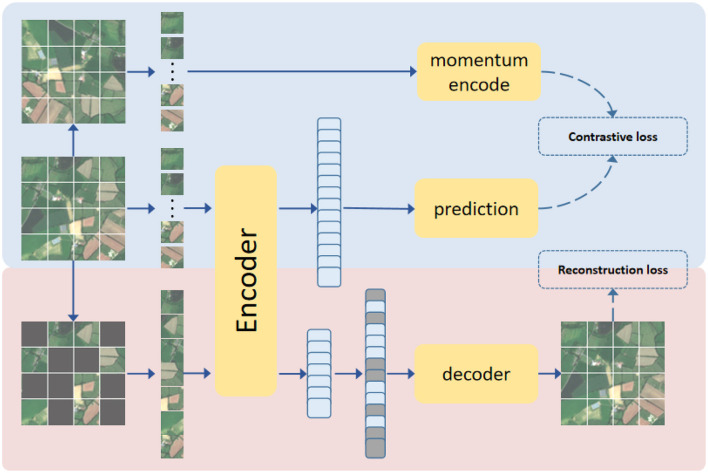
The SSL algorithm structure where the encoder is trained based on the MAE algorithm and the MoCoV3 algorithm.

The overall structure of the self-supervised learning model used in this paper is shown in Figure 5. Algorithm 1 provides the pseudo-code of loss for this pretext task. We have verified that a pre-trained encoder that combines both SSL model structures performs better than a pre-trained encoder with a single structure.

**Algorithm 1:**
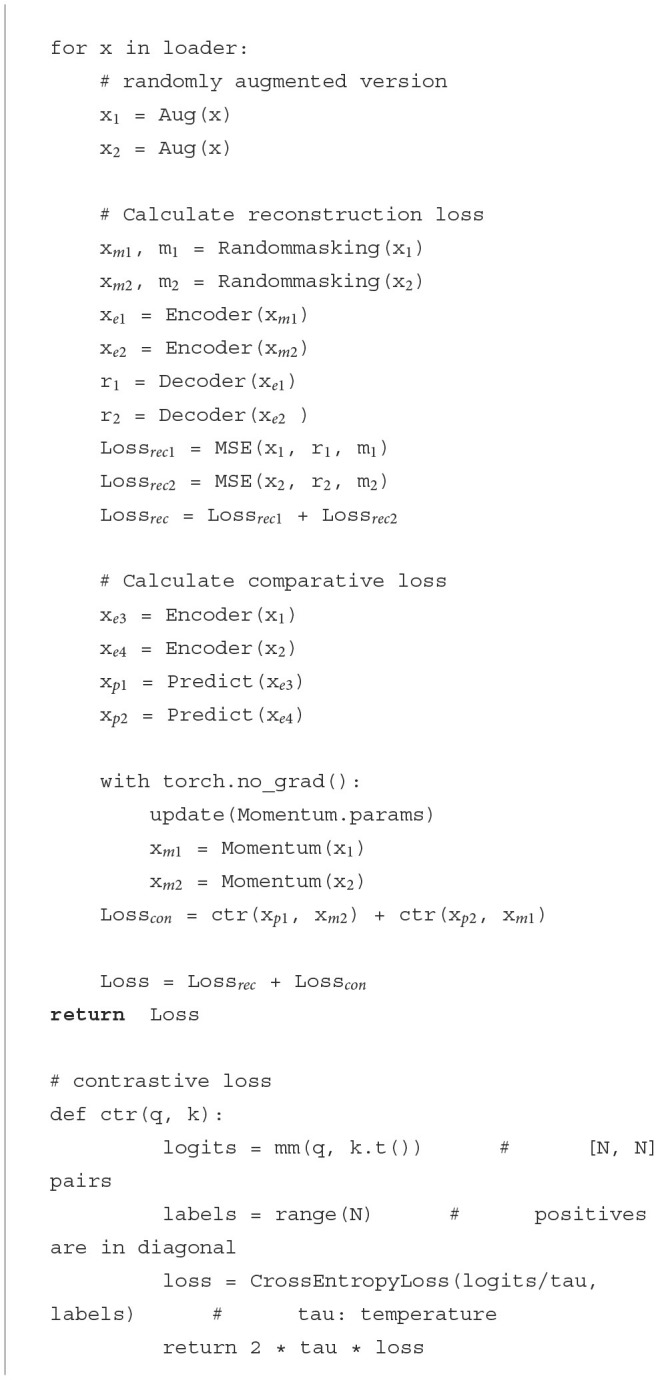
Pseudocode of loss function in a PyTorch-like style.

## 4 Experimental results

### 4.1 Datasets

**Figure 6:**
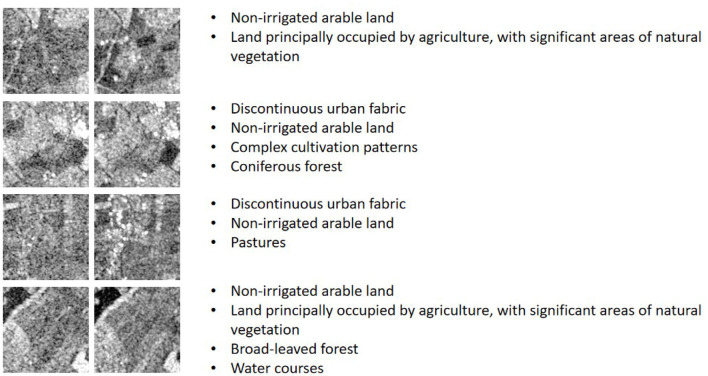
S1 Data visualization and corresponding multi-label information.

**Figure 7:**
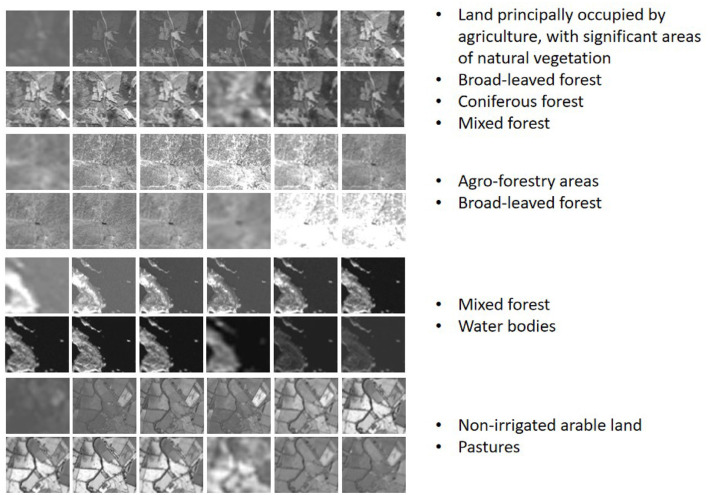
S2 Data visualization and corresponding multi-label information.

In this paper, we use BigEarthNet-MM (Sumbul et al., 2021) dataset for both self-supervised training and classification task evaluation, which is the most common multi-label scene classification dataset in remote sensing so far. We use PyTorch and Adam optimizer to train our network. The GPU and CPU configuration of the computer are NVIDIA GeForce RTX 3090 and Intel(R) Xeon(R) Gold 6226R CPU, respectively. BigEarthNet is a benchmark dataset that consists of 590,326 non-overlapping Sentinel-1 (S1) and Sentinel-2 (S2) image patches acquired between June 2017 and May 2018 over the 10 European countries, S1 patches were enriched with Synthetic Aperture Radar and S2 patches were enriched with spectral bands at 10, 20, and 60-m resolution. For S1, two channels are available, and for S2, twelve channels are available. Each image patch is annotated by multiple land-cover classes (i.e., multi-labels) taken from the CORINE Land Cover database of the year 2018 (CLC 2018). Originally, 43 labels were used. These were later merged into 19 labels. We show some samples of S1 data and S2 data respectively in Figures 6, 7, and carry out normalized visual processing display. As can be seen from the visual images of S1 and S2, the data of SAR and MS modes are still very different. SAR data mainly provides the physical characteristics of the ground and reflects the surface roughness of the ground objects, making it difficult to interpret the image content. However, MS data is easy to interpret and helps to identify various types of ground objects. So we use both kinds of data together.

We divided the data set into 311,667 training sets, 103,944 validation sets and 118,065 test sets, where the data covered by snow or cloud cover was dropped. Since the BigEarthNet-MM images are 120 × 120 pixels, we resize all samples to 224 × 244. We perform self-supervised pre-training on the training set without labels and fine-tune the classification task using 19 labels in the downstream task.

### 4.2 Shortcut number evaluation

**Figure 8:**
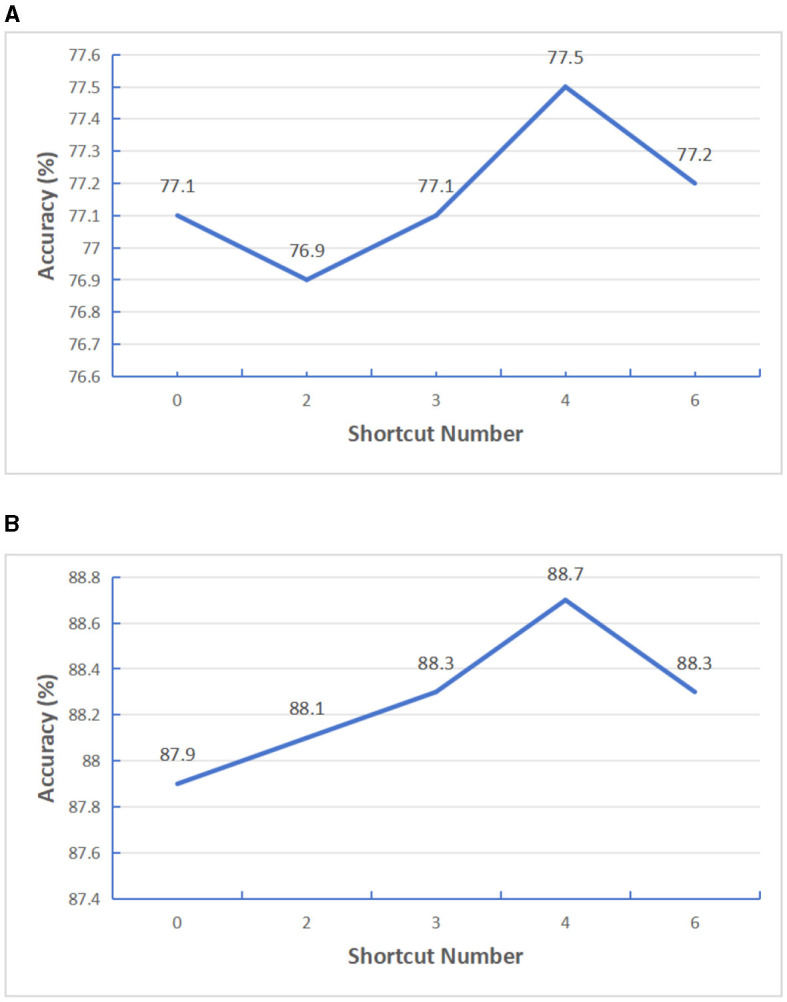
Testing the impact of varying shortcut layers with encoder depth set at 12. **(A)** S1 patches. **(B)** S2 patches.

**Figure 9:**
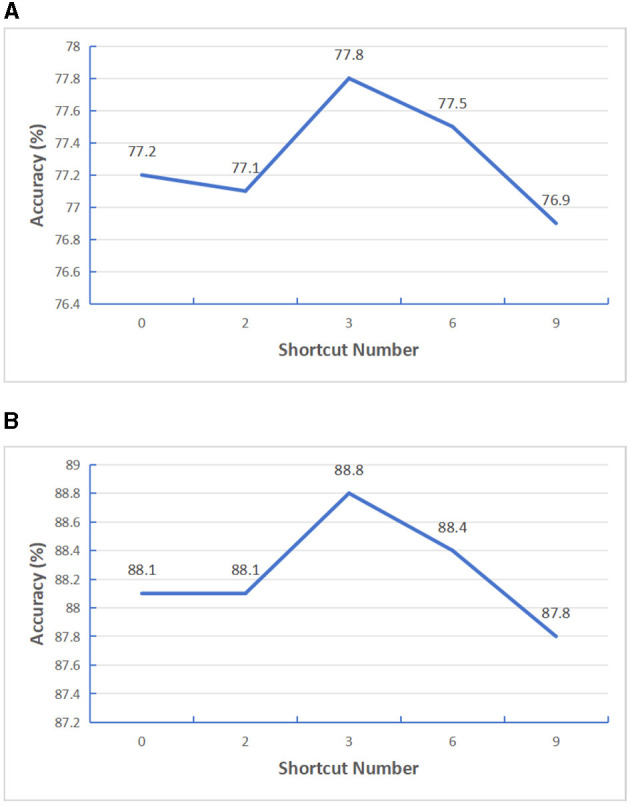
Testing the impact of varying shortcut layers with encoder depth set at 18. **(A)** S1 patches. **(B)** S2 patches.

Firstly, we improve the transformer model structure. Considering that features extracted from shallow transformer will be forgotten to a certain extent with the deepening of model depth, in order to effectively use the effective information extracted from shallow transformer, we added a gating unit after N transformers layers to fuse the previous feature information. In order to verify how many transformers layers in the encoder structure can get the best performance, we added a gating fusion on the basis of the SatViT (Fuller et al., 2022) model structure. We first set the Encoder depth as 12 layers, select the number of shortcut layers *N* as 0, 2, 3, 4, 6, and conduct supervised training tests based on BigEarthNet-S1 data and BigEarthNet-S2 data, The test results are shown in Figure 8. It can be seen from the results that the best result can be obtained when gated fusion is carried out by four transformers. Similarly, in order to verify the effect of other Encoder depths, we selected Encoder depths of 18 for testing and the number of shortcut layers *N* as 0, 2, 3, 6, 9, respectively, and conducted supervised training tests based on BigEarthNet-S1 data and BigEarthNet-S2 data respectively. The test results are shown in Figure 9, it can be seen from the results that the best results can be obtained when gated fusion is carried out by the three transformers. Therefore, we believe that layer hopping connections for multiple transformer layers can improve the feature extraction capability of Encoder, but the number of layers is not too large. In later experiments, we uniformly used models with Encoder depth 12.

### 4.3 Multi-modal data fusion evaluation

Since remote sensing data usually contains multi-modal data, the BigEarthNet-MM we used generally contains multi-modal data. Recently, multi-modal fusion has become a research hotspot of everyone. However, we found that many BigEarthNet studies simply spliced S1 and S2 data. Because S1 patches were enriched with Synthetic Aperture Radar and S2 patches were enriched with spectral bands at 10, 20, and 60-m resolution. We believe that data of different modes should have different proportions in the feature extraction process, so we propose a GMU structure to fuse the two modal data. In order to verify our method, we conduct four sets of experiments based on the MGSViT model, which are (1) S1 data single-modal supervised training; (2) S2 data single-mode supervised training; (3) Simple spliced together with S1 and S2 data for supervised training; (4) S1 data and S2 data are supervised train through gated fusion training. The test results are shown in Table 1. It can be seen from the results that the two kinds of data are best fused by gating.

**Table 1:** Impact of data fusion on model training results (%) with different data modal, the values marked in bold indicate that model is the best performer in the corresponding item.

**Datasets**	**S1**	**S2**	**S1 + S2**	**S1 GMU S2**
Accuracy	77.1	87.9	88.2	**88.9**

**Figure 10:**
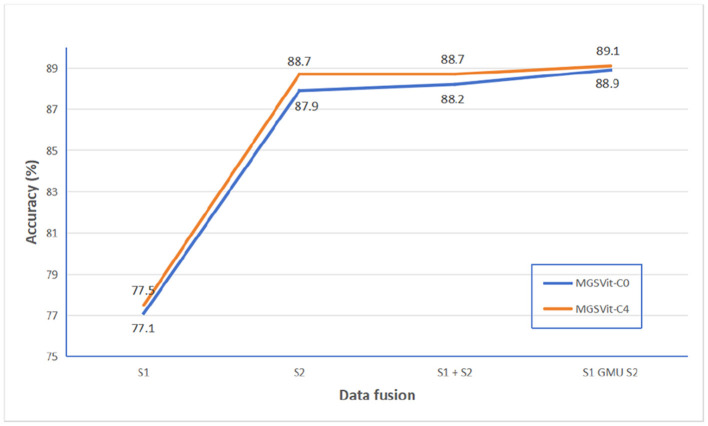
Results of MGSViT-C0 model and MGSViT-C4 model in different modal data fusion modes.

At the same time, we compared the test results of MGSViT model without shortcut layers structure(MGSViT-C0) and MGSViT model with 4 shortcut layers structure (MGSViT-C4) under different fusion modes of single mode data and multi-mode data. The test results are shown in Figure 10. The results show that MGSViT-C4 model structure is still the best in S1 and S2 multi-mode gated fusion.

### 4.4 SSL encoder evaluation

**Figure 11:**
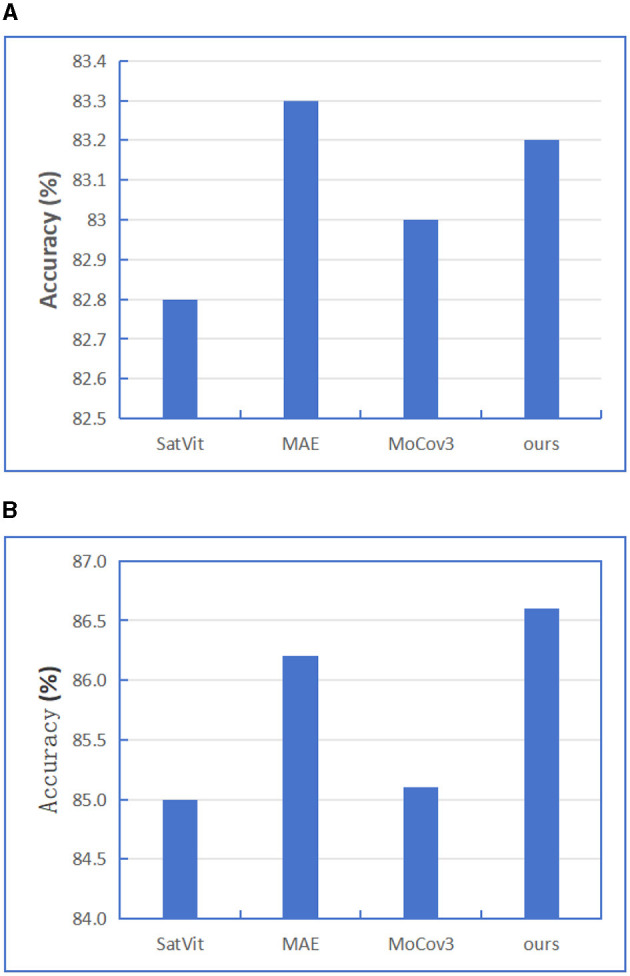
BigEarthNet-S2 performance depending on different SSL model structures to train downstream task, We report linear probing, fine-tuning results. **(A)** SSL + Linear. **(B)** SSL + Fine-tune.

As we all know, self-supervised learning has the ability to learn common expressions from large-scale, unlabeled data, and Transformers structure has also achieved good results in the field of self-supervised learning. Therefore, in order to further improve the feature extraction capability of model encoder, we refer to the contrast learning idea in the self-supervised learning algorithm. The momentum contrast (MoCov3) method is introduced on the basis of MAE model. First, BigEarthNet-MM data is used to self-supervise the training of Encoder part, and then the pre-trained Encoder model is transferred to the supervised downstream task. When designing the self-supervised training model, we referred to the training ideas of MAE and MoCov3 models on the basis of SatViT model, so we tested SatViT, MAE, MoCov3 and our designed model without shortcut layers for a fair comparison respectively, and only selected BigEarthNet-S2 data for testing. When self-supervised training encoders and decoders, We apply several image augmentation methods including RandomResizedCrop with cropping scale ranges from 20% to 100% of the original image size. RandomBrightness/Contrast randomly applies either random brightness adjustment or random contrast adjustment to the image with a probability of 80%. The random brightness adjustment has a maximum delta of 0.4, and the random contrast adjustment has a maximum delta of 0.4. RandomGrayscale converts the image to grayscale with a probability of 20%. RandomGaussianBlur with a probability of 100%. The blur radius is randomly chosen from a range between 0.1 and 2. And We set the same epochs and optimizer. After the pre-training, we perform commonly used linear probing (freezing the pre-trained encoder) and fine-tuning for the downstream tasks. The test results are shown in Figure 11. From the results figure, we find in SSL and linear experiments, although our results are lower than MAE but the difference is not large, and in SSL and fine-tuning experiments, our results are the best, which is 0.4 higher than MAE algorithm. The results show that the self-supervised training method designed by us is generally better than the current mainstream self-supervised learning methods.

### 4.5 Ablation studies

In order to analyze the influence of each improvement point on the performance of the model, we supplemented the ablation experiment of each improved module on the basis of the above test experiment, In detail, We compared different versions defined on the basis of six different configurations. The experiments with SSL module adopted the strategy of self-supervised training and fine-tuning the whole model structure, while the other experiments without SSL module adopted the strategy of supervised training. The results of the ablation experiment were summarized in the Table 2.

**Table 2:** Ablation of model baselines.

**Case**	**Shortcut**	**Multi-modal fusion**	**SSL**	**Accuracy (%)**
MGSViT-NO				88.2
MGSViT-C4	✓			88.7
MGSViT-C0-GMU		✓		88.9
MGSViT-C4-GMU	✓	✓		89.1
MGSViT-SSL			✓	88.3
MGSViT-base	✓	✓	✓	90.1

MGSViT-NO: the model structure without shortcut layers, simply splice multiple modal data and supervised training.

MGSViT-C4: the model structure with four shortcut layers, simply splice multiple modal data and supervised training.

MGSViT-C0-GMU: the model structure without shortcut layers, gated fusion of multi-modal data and supervised training.

MGSViT-C4-GMU: the model structure with four shortcut layers, gated fusion of multi-modal data and supervised training.

MGSViT-SSL: the model structure without shortcut layers, simply splice multiple modal data and self-supervised learning training.

MGSViT-base: the model structure with four shortcut layers, gated fusion of multi-modal data and self-supervised learning training.

According to the results of ablation experiments, one can observe that the intra-modal shortcut connection, inter-modal multi-modal fusion and self-supervised training models proposed by us can effectively improve the performance of the models. In detail, the intra-modal shortcut connection can improve model performance by 0.5, gated multi-modal fusion can improve model performance by 0.7, self-supervised learning can improve model performance by 0.1, and the standard base model can improve model performance by 1.9 with the above improvements.

### 4.6 Comparison with previous results

In this sub-section, we evaluate the effectiveness of the proposed method and the most advanced methods in the field of remote sensing on BigEarthNet-MM datasets. In order to verify the validity of our proposed method, we use the model structure with 4 shortcut layers and gated fusion of multi-modal data, so we use BigEarthNet-S1 data and BigEarthNet-S2 data to self-supervised train two Encoder models, respectively. When self-supervised training encoders and decoders, the input data size for the model is set to 224 × 244. We run 100 epochs of end-to-end training on each mode using the Adam-W optimizer, and a max learning rate of 1*e*^−5^ decreasing to 0 according to a cosine schedule. Similarly, we designed the downstream task for comparison experiment. Our downstream task is multi-label image classification. During the training of the downstream classification task, we fine-tuned the parameters of the entire model, also set the input size to 224 × 244, and 100 epochs of end-to-end training for each mode with Adam-W optimizer. and a max learning rate of 1*e*^−4^ decreasing to 0 according to a cosine schedule. We use a self-supervised pre-trained model to initialize the encoder parameters while randomly initializing the task classification headers. The decoder of the pre-trained model does not participate in downstream tasks.

We compared the most advanced multi-modal classification models, DINO-MM (Wang et al., 2022a) and MoCo-MM (Wang et al., 2022b) on the dataset BigEarthNet-MM. In the DINO-MM, they report random initialization, self-supervised pre-training joint SAR-optical pre-training (DINO-MM), and fully supervised learning. In the MoCo-MM, they integrate SAR data by early fusion, and use RandomSensorDrop as an additional data augmentation strategy, and the model gets fed random combinations of SAR/optical patches, thus learning both inner- and inter-modality representations. Then they compare multi-modal pre-training (MM) to uni-modal pre-training (S1/2) on BigEarthNet. We conducted experiments on self-supervised pre-training with fine-tuning of downstream tasks and supervised training respectively, and conducted comparative experiments on single mode and multi-mode. The experimental results are shown in Table 3.

**Table 3:** Linear classification results (%) on the BigEarthNet-MM dataset.

**Model**	**Method**	**S1**	**S2**	**S1 + S2**	**S1 GMU S2**
DINO-MM	SSL + fine-tune	79.5	87.1	87.1	–
DINO-MM	Supervised	77.1	86.7	88.6	–
MoCo-MM	SSL + fine-tune	79.5	85.1	85.2	–
MoCo-MM	Supervised	77.2	88.7	88.9	–
MGSViT (ours)	SSL + fine-tune	80.3	88.9	89.1	90.1
MGSViT (ours)	Supervised	77.5	88.7	88.7	89.1

It can be seen from the results that the proposed method is superior to the most advanced methods in terms of both self-supervised training and supervised training. In detail, in the single-mode experiments of S1 and S2, our results are generally higher than those of DINO and MoCov3 methods. In the S1 + S2 experiments, the supervised training results of our model are not much different from those of DINO and MoCov3 methods, but the results of SSL + fine-tune are superior, 2% higher than the DINO method result and 3.9% higher than the MoCov3 method result. In the S1 GMU S2 experiment, only we used this method, and it can be observed that the multi-modal data fusion method is better than the direct splicing method, and the result can reach 90.1% after adding SSL and fine-tune.

## 5 Conclusion

In this paper, a new multi-modal gated fusion self-supervised training method is proposed for image classification in remote sensing field. The proposed method extracts multi-modal feature representations by means of intra-modal shortcut gated fusion and inter-modal feature gated fusion, and uses a new self-supervised training method to learn encoder module. In detail, we have designed the intra-modal and inter-modal gated fusion. In the internal structure of the encoder, a series of transformers are stacked, and we have designed a layer hopping mechanism. We have learned through experiments that when the total depth of the Encoder is 12 layers, gating fusion every four layers of transformers has the best effect, which can extract the shallow and deep features of the data more effectively. If the Encoder is of other depths, the number of layers of the shortcut layer connection will be different. After the feature representation is extracted from encoder structure, the features of different modes are gated and fused to obtain the effective feature representation based on different modes. We have tested two modes fusion methods, one is directly splicing the feature vectors of two modes, and the other is controlling the fusion proportion of each mode feature through a gating system. Through the test and comparison, we find that it is better to control multi-mode fusion by gating system. In the self-supervised training model, we conducted self-supervised pre-training of different modal structures based on mask reconstruction self-supervised method and momentum contrast self-supervised method, and then fine-tuned the trained encoder for subsequent downstream classification tasks. Through experiments, we compared the effects of the reconstruction method alone, the momentum contrast method alone and the combination of the two methods. It is found that the combination of the two methods can extract data features more effectively, and the generated Encoder model has stronger representation ability.

Finally, we conducted a series of experiments on the remote sensing open data set BigEarthNet, including verifying the impact of each module on the model performance, and comparing SSL + fine-tune and self-supervised training experiments with the most advanced published methods. Experimental results demonstrate the effectiveness of the proposed multi-modal gated fusion self-supervised training method, and prove that the proposed method is superior to the most advanced methods.

## Data Availability

The original contributions presented in the study are included in the article/supplementary material, further inquiries can be directed to the corresponding author.
