# Correction: Multi-label remote sensing classification with self-supervised gated multi-modal transformers

**DOI:** 10.3389/fncom.2025.1665406

**Published:** 2025-08-18

**Authors:** Na Liu, Ye Yuan, Guodong Wu, Sai Zhang, Jie Leng, Lihong Wan

**Affiliations:** ^1^University of Shanghai for Science and Technology, Institute of Machine Intelligence, Shanghai, China; ^2^Origin Dynamics Intelligent Robot Co., Ltd., Zhengzhou, China

**Keywords:** self-supervised learning, pre-training, vision transformer, multi-modal, gated units

In the published article, there was an error in the Funding statement. The Funding statement was erroneously omitted, and financial support grants should have instead been included. The correct Funding statement appears below.

